# An Integrative Heterogeneous Graph Neural Network–Based Method for Multi-Labeled Drug Repurposing

**DOI:** 10.3389/fphar.2022.908549

**Published:** 2022-07-06

**Authors:** Shaghayegh Sadeghi, Jianguo Lu, Alioune Ngom

**Affiliations:** School of Computer Science, University of Windsor, Windsor, ON, Canada

**Keywords:** computational drug repurposing, graph embedding, graphsage, data integration, link prediction, graph neural network

## Abstract

Drug repurposing is the process of discovering new indications (i.e., diseases or conditions) for already approved drugs. Many computational methods have been proposed for predicting new associations between drugs and diseases. In this article, we proposed a new method, called DR-HGNN, an integrative heterogeneous graph neural network-based method for multi-labeled drug repurposing, to discover new indications for existing drugs. For this purpose, we first used the DTINet dataset to construct a heterogeneous drug–protein–disease (DPD) network, which is a graph composed of four types of nodes (drugs, proteins, diseases, and drug side effects) and eight types of edges. Second, we labeled each drug–protein edge, *dp*_*i*,*j*_ = (*d*_*i*_, *p*_*j*_), of the DPD network with a set of diseases, {*δ*_*i*,*j*,1_, … , *δ*_*i*,*j*,*k*_} associated with both *d*_*i*_ and *p*_*j*_ and then devised multi-label ranking approaches which incorporate neural network architecture that operates on the heterogeneous graph-structured data and which leverages both the interaction patterns and the features of drug and protein nodes. We used a derivative of the GraphSAGE algorithm, HinSAGE, on the heterogeneous DPD network to learn low-dimensional vector representation of features of drugs and proteins. Finally, we used the drug–protein network to learn the embeddings of the drug–protein edges and then predict the disease labels that act as bridges between drugs and proteins. The proposed method shows better results than existing methods applied to the DTINet dataset, with an AUC of 0.964.

## 1 Introduction

Drug repurposing (DR) is a process of identifying novel therapeutic purposes for existing drugs. Over the years, computational drug repurposing (CDR), known as *in silico* drug repurposing, has gained considerable popularity in the pharmaceutical industry due to its time and cost efficiency in the drug development process compared to the traditional *de novo* drug discovery process. Drug repurposing can be a promising treatment strategy for a lot of health crises such as COVID-19 since it can shorten the drug development process with much less funding (Sadeghi et al., 2021; Su et al., 2021). In recent years, different computational approaches are suggested for repurposing drugs based on machine learning, network analysis, and text mining (Li et al., 2016). Since network-based methods are capable of using ever-increasing large-scale biological datasets such as genetic, pharmacogenomics, clinical, and chemical data, they are more desirable for drug repurposing tasks (Sadeghi and Keyvanpour, 2019).

With the recent advances in deep learning methods on graphs due to their promising ability to capture complex and highly non-linear network structures, graph neural network (GNN) method usage on biological networks seems more interesting than ever (Pan et al., 2022), (Yu J.-L. et al., 2021). For example, Yu Z. et al. (2021) proposed a layer attention graph convolutional network (LAGCN) for the drug–disease association prediction. The LAGCN utilizes a GCN to capture structural information from the heterogeneous network composed of drug–disease associations, drug–drug similarities, and disease–disease similarities. The attention mechanism is introduced to combine the embeddings from different convolution layers, which leads to a more informative representation of drugs and diseases. Wang et al. (2020) proposed BiFusion, a bipartite GCN model for CDR through heterogeneous information fusion. BiFusion combines insights from multiscale pharmaceutical information by constructing a multi-relational graph of drug–protein, disease–protein, and protein–protein networks. (Cai et al. (2021) proposed a method, called DRHGCN, based on the heterogeneous information fusion graph convolutional network. deepDR, on the other hand, uses a variational auto-encoder (VAE) to infer candidates for repurposing (Zeng et al., 2019). Zhao et al. (2021) proposed a method called multi-graph representation learning (MGRL), which first uses the graph convolution network to learn the graph representation of drugs and diseases. Then, the graph embedding algorithm represents the relationships between drugs and diseases. Finally, the two kinds of graph representation learning features were put into the random forest classifier for training. The drug repositioning method based on heterogeneous networks and text mining (HeTDR) proposed by Jin et al. (2021) fuses network topology information and text mining information to gain and predicts potential drug-disease associations by an embedding learning method.

The main difference between these aforementioned deep learning-based methods for CDR tasks is that they use different types of network inputs or add extra features and also different GCN structures as decoders. Hence, one way to expand these methods is to include additional biological network types in the equation of the DR task. However, creating the base heterogeneous network for CDR is a challenging task.

This study casted the CDR problem as a link prediction task and proposed DR-HGNN, a novel approach for inferring new drug-disease associations (i.e., new links between drugs and diseases). The main idea is to create a multi-label heterogeneous drug–protein–disease (DPD) network as input for the heterogeneous variation of the GraphSAGE algorithm.

First, DR-HGNN integrates six heterogeneous networks and four homogeneous networks for creating drug and protein side information, which can potentially improve the performance of CDR. Second, DR-HGNN creates a DPD network in which, for each drug and protein in the drug–target interaction (DTI) network, we assume there is at least one disease that connects these two. In other words, we used diseases as our labels in the DTI network. However, this leads to a multi-label problem which means that there can be more than one disease as a bridge for each DTI. Hence, in the third step, we solved this problem with a transformation-based solution. Later, we used a generalized version of GraphSAGE for heterogeneous networks, called HinSAGE. HinSAGE processes the input DPD network for embedding each drug and protein node. Finally, an edge embedding layer will be used for predicting new disease edges between drugs and proteins. This edge embedding scores each edge between drug and proteins and the disease label associated with this edge. DR-HGNN shows a high predictive performance when compared to existing CDR methods.

## 2 Methods

Algorithm 1 DR-HGNN

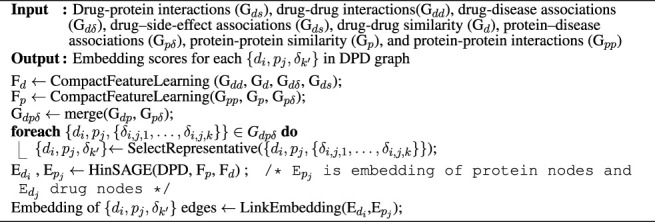

### 2.1 Construction of the DPD Graph

**FIGURE 1:**
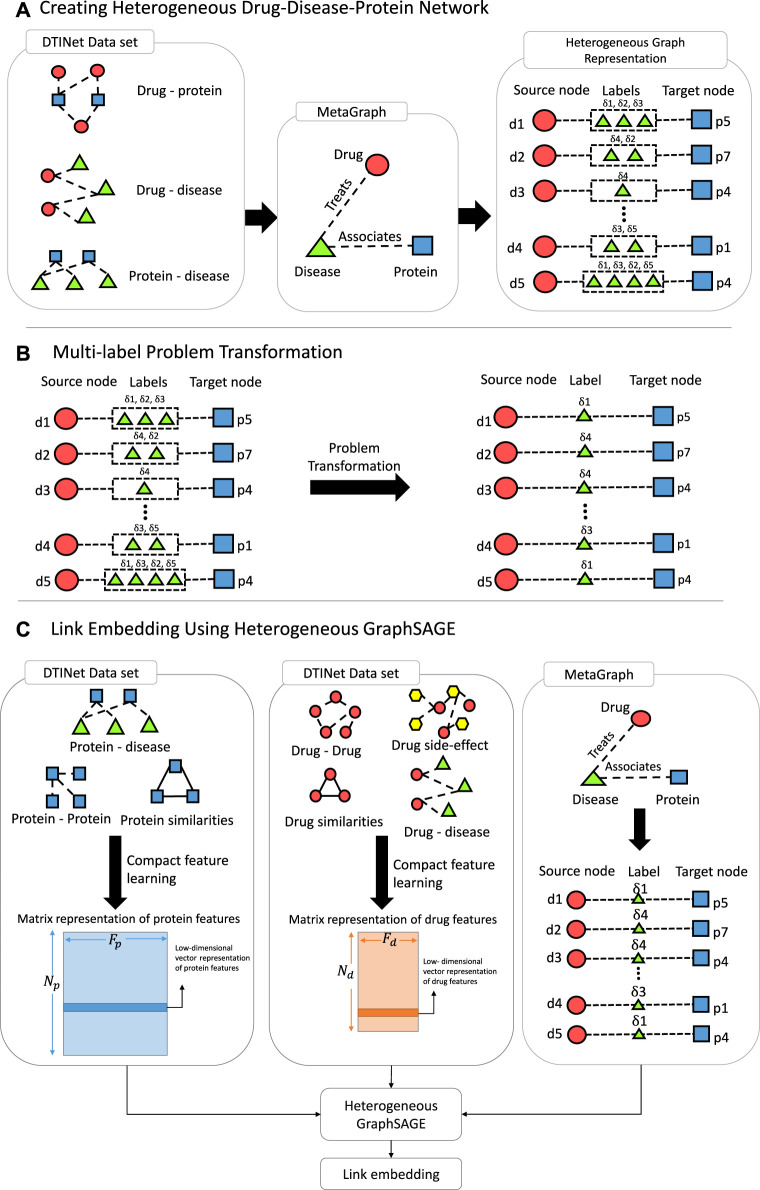
Pipeline of DR-HGNN. **(A)** Creating Heterogeneous Drug-Disease-Protein Network: using the DTINet dataset, a meta-graph is created, which can be presented as a Heterogeneous Graph (on the right). **(B)** Multi-label Problem Transformation: A problem transformation technique is used since the Heterogeneous Graph from step A is multi-labeled. **(C)** Link Embedding Using Heterogeneous GraphSAGE: With matrix representation of each protein and drug and the heterogeneous graph from step B, Heterogeneous GraphSAGE embeds links between nodes of this heterogeneous graph.

We constructed a schema of a DPD heterogeneous graph (Figure 1A). Diseases in this graph are bridges between drugs and proteins; they are labels on edges connecting drugs and proteins. This graph is constructed from three different heterogeneous sub-networks (i.e., drug–protein interaction, protein–disease association, and drug–disease association).

Each edge in this graph connects drugs and proteins and has diseases as their label. This means we can have a triple of (drugs, proteins, and diseases) for each link {*d*_*i*_, *p*_*j*_, {*δ*_*i*,*j*,1_, … , *δ*_*i*,*j*,*k*_}}. Each drug and protein has a feature vector constructed by a compact feature learning method (Luo et al., 2017). The input for the compact feature learning method is different types of sub-networks (i.e., drug–protein interactions (G_*ds*_), drug–drug interactions (G_*dd*_), drug–disease associations (G_*dδ*_), drug–side-effect associations (G_*ds*_), protein–disease associations (G_*pδ*_), protein–protein similarity (G_*p*_), drug–drug similarity (G_*d*_), and protein–protein interactions (G_*pp*_)) that have side information from a different view of each entity (i.e., drugs and proteins). The output of the compact feature learning method is a matrix representation of the entity (i.e., drugs and proteins) features. The compact feature learning method integrates diverse information from the heterogeneous network by foremost combination of the network diffusion algorithm (random walk with restart) with a dimensionality reduction technique (diffusion component analysis) to obtain informative but low-dimensional vector representations of nodes in the network (Luo et al., 2017).

Finally, based on drug–protein and protein–disease associations, we labeled the edges the in drug–protein interaction network to create our DPD graph (*G*_*dpδ*_).

$$G_{dp\delta }\leftarrow merge\left(G_{dp},G_{p\delta }\right),$$ (1)

where merge joins adjacency matrices of *G*_*dp*_ and *G*_*pδ*_. Here, the joining is carried out on protein names. The G_*dpδ*_ graph constructed here can be presented as the triple of (drugs, proteins, and diseases) {*d*_*i*_, *p*_*j*_, {*δ*_*i*,*j*,1_, … , *δ*_*i*,*j*,*k*_}}.

### 2.2 Multi-Label Problem Transformation

The constructed G_*dpδ*_ has multi-label edges (Figure 1B). Not all labels are equally important to the characterization of the edges. Hence, we need a multi-label ranking approach to choose just one of the labels as the labels’ representative. One-hot encoding of labels in the G_*dpδ*_ graph can be sparse and large. Hence, instead of using a neural network for dealing with our multi-labeled edges, we proposed a method as a prepossessing step to transform our multi-label problem into a single-label problem (Tsoumakas et al., 2009). This method transforms the multi-label learning task into a multi-class or single-label classification task. In other words, LP models the joint distribution of labels. It treats each label subset in the multi-label training set as a class of a multi-class task, and the prediction will be one of these subsets (Chu et al., 2021). For this purpose, for each set of labels for each pair of drug–protein, we used one of the labels as the representative of that set.

For selecting this representative label, we selected the label with less frequency among all sets of labels and more mutual information. This representative label is more informative than other labels since these labels have a more distinctive ability based on the law of frequency. This new DPD network is a compressed version of G_*dpδ*_, and every link in this graph can be also presented as unique {*d*_*i*_, *p*_*j*_, *δ*_*k*′_}. For this purpose, first, we counted the frequency of each disease in the G_*dpδ*_ graph and then we selected one disease for each pair of drugs and proteins that has the least appearance in the network. This disease is the new label for the drug–protein association.

### 2.3 Edge Embedding Using Heterogeneous GraphSAGE

Standard message passing GNNs cannot trivially be applied to heterogeneous graph data as the same functions cannot process node and edge features from different types due to differences in the feature type and size (Fey and Lenssen, 2019). To avoid this problem, here, we used a generalized version of the GraphSAGE algorithm (Hamilton et al., 2017) for heterogeneous graphs called HinSAGE (Data61, 2018).

Looking at Table 1, HinSAGE can provide us with the features we want from our GNN, while other methods such as the graph attention network (GAT), graph convolutional network (GCN), and GraphSAGE cannot be performed on heterogeneous networks without implementing message and update functions individually for each edge type.

**TABLE 1:** Comparison of GNN methods.

Method	Handle bipartite graph	Handle heterogeneous graph	Handle different node feature sizes
HinSAGE	Yes	Yes	Yes
GraphSAGE	Yes	No	Yes
GCN	No	No	No
GAT	Yes	No	Yes

HinSAGE separate neighborhood weight matrices (*W*_neigh_’s) for every unique ordered tuple of (*N*_1_, *E*, *N*_2_) where *N*_1_ and *N*_2_ are node types (here, *N*_1_ is for drugs, and *N*_2_ is for proteins), and *E* is an edge type (here, *E* is for disease) to support heterogeneity of nodes and edges. HinSAGE also will distinct self-feature matrices *W*_self_ for every node type, where *W*_self_ is a unique self-edge type for every node type.

As for feature update rules, aggregation (mean) of features from the neighbors of node *v* *via* edges of type *r* is being used:

$$h_{N_{r}\left(v\right)}^{k}=\frac{1}{N_{r}\left(v\right)}\underset{\begin{array}{c} u\in N_{r}\left(v\right) \end{array}}{\sum }D_{p}\left[h_{u}^{k-1}\right].$$ (2)

(Data61, 2018).

Meanwhile, forward pass through layer k is as follows:

$$h_{v}^{k}=\sigma \left(W_{t_{v},self}^{k}D_{p}\left[h_{v}^{k-1}\right]+W_{r,self}^{k}h_{N_{r}\left(v\right)}^{k}+b^{k}\right).$$ (3)

(Data61, 2018).

Here, $W_{t_{v},self}^{k}$ is the weight matrix for self-edges for node type *t*_*v*_ and is of shape *d*_*k*_ × *d*_*k*−1_. Also, $W_{r,self}^{k}$ is the weight matrix for edges of type *r* and is of shape $\frac{d_{k}}{2}\times d_{k-1}\left(r\right)$ . *D*_*p*_ [.] is a random dropout with probability *p* applied to its argument vector. *σ* is the nonlinear activation. *b*_*k*_ is an optional bias, while $h_{v}^{k}$ is the output for node *v* at layer *k*. *r* indicates the edge type from node *v* to node *u* (*r* is defined as the unique tuple (*t*_*v*_, *t*_*e*_, *t*_*u*_)), where *t*_*v*_ indicates the type of node *v*, and *t*_*e*_ indicates the relation type. *N*_*r*_(*v*) is a neighbor of the node *v* *via* the edge type *r*. $d_{k-1}\left(r\right)=\dim\left(h_{N_{r}\left(v\right)}^{k}\right)$ is the dimensionality of (*k* − 1)-th layer’s features of node *v*’s neighbors *via* edge type *r*. The number of trainable parameters per layer *k* for this model is as follows:

$$T_{v}d_{k}d_{k-1}+R_{e}d_{k}d_{k-1}+d_{k}=\left(T_{v}+R_{e}\right)d_{k}d_{k-1}+d_{k},$$ (4)

(Data61, 2018). supposing that the dimensionalities of all destination node features for all edge types *r* are all equivalent, that is, *d*_*k*−1_(*r*) = *d*_*k*−1_ *∀r*, the number of all node types in the graph is *T*_*v*_, and the number of all edge types is *R*_*e*_.

The HinSAGE algorithm requires two types of input: node features and an adjacency matrix of the heterogeneous graph. Here, drugs and proteins are our nodes, and we used the compact feature learning method to obtain their feature vectors. Compact feature learning is a random walk-based algorithm. First, a random walk algorithm with restart (RWR) is used to compute the diffusion states of individual networks. Then, the low-dimensional representations of feature vectors for each node are obtained by minimizing the difference between the diffusion states *s*_*i*_ and the parameterized multinomial logistic models ${\hat{s}}_{i}$ . The low-dimensional feature vectors obtained from the previous step encode the relational properties (for example, similarity), association information, and topological context of each node in the heterogeneous network (Luo et al., 2017). As for the adjacency matrix of the heterogeneous graph, we constructed a DPD graph in Section 2.1.

After having embeddings of each node, we can use a function that predicts a multi-class edge classification output from (source: drug; destination: protein) node embeddings (node features). For this purpose, this layer combines (source: drug; destination: protein) new embeddings from HinSAGE layers into edge embeddings.

## 3 Methodology

This section demonstrates the efficacy and efficiency of the DR-HGNN frameworks for DR tasks and compares them with three state-of-the-art DR algorithms. Five aspects are discussed in the following four subsections: datasets in both our proposed model and the competing models, experiment setting, results of competing methods, and case studies of our proposed model.

### 3.1 Material and Data

In this study, the DTINet data set from Luo et al. (2017) is used. DTINet is a heterogeneous network with 12,015 nodes and 1,895,445 edges in total and is originally constructed for predicting missing DTI (drug–target interaction) edges. Luo et al. (2017) compiled various curated public drug-related databases (DrugBank (Wishart et al., 2006), the Comparative Toxicogenomics Database (CTD) (Davis et al., 2019), the Human Protein Reference Database (HPRD) (Keshava Prasad et al., 2009), and Side Effect Resource (SIDER) (Kuhn et al., 2010)) (Table 2) to create DTINet. The DTINet network integrates four types of nodes (that is, drugs, proteins, diseases, and drug side effects) and six types of edges (that is, drug–protein interactions, drug–drug interactions, drug–disease associations, drug–side effect associations, protein–disease associations, and protein–protein interactions).

**TABLE 2:** Number of nodes and edges of individual types in the constructed heterogeneous network on DTINet (Luo et al., 2017).

Node	Number of edges			
	Drug	Protein	Disease	Side effect
Drug	10, 036	1, 923	199, 214	80, 164
Protein	1, 923	7, 363	1, 596, 745	–
Number of nodes	708	1, 512	5, 603	4, 192

Based on chemical structures of drugs and primary sequences of proteins, they also built multiple similarity networks to further augment the network heterogeneity, providing diverse information from a multiple-view perspective. The heterogeneous DPD graph has only 1,923 triples of {*d*_*i*_, *p*_*j*_, *δ*_*k*_} constructed by the DTINet data set. As shown in Table 2, the DPD graph has 708 drug nodes and 1,512 protein nodes with 39 diseases, representing 5,603 diseases selected in Section 2.2.

### 3.2 Experimental Setup

CDR can be cast as a link prediction problem, and here, in this study, we predicted the edges between drugs and proteins with the diseases as their label. To evaluate the prediction performance of the DR-HGNN model and the competing methods, we used 5-fold cross-validation (5-CV) since other baseline methods also used 5-CV. We added a matching number of non-interacting triples to the known interacting drug–target–disease triples (DPD graph). Then, data sets were five times shuffled to form five randomly ordered data sets, each of which was divided into training (60%), validation (20%), and test sets (20%) (Luo et al., 2017; Moon et al., 2021).

In experiments, the area under the receiver operating characteristic curve (AUC-ROC) and precision-recall curve (AUPR) are used to measure the performance of results. AUC-ROC and AUPR, as useful measures of accuracy, have been considered, with a meaningful AUC interpretation usually representing the overall performance of the method (Sadeghi and Keyvanpour, 2019). We compared our approach with five other DR methods in the following:

• **DTINet** (Luo et al., 2017), is a low-dimensional vector representation-based method that extracts features from the topology of the nodes in the integrated network and predicts and computes drug–protein target interactions and drug similarity measures through these representations.
• **NMTF** (Ceddia et al., 2020), is a negative matrix factorization-based method that imposes a non-negative constraint on the factorized matrices during multiplication and update operations.
• **LAGCN** (Yu Z. et al., 2021), is a layer attention graph convolutional network-based method for the drug–disease association prediction.
• **deepDR** (Zeng et al., 2019), is an autoencoder-based method for fusing the features and mining new drug disease associations.
• **KBMF** (Gönen et al., 2013), is a kernelized bayesian matrix factorization method that can make use of multiple side information sources and can be applied in recommender systems.

### 3.3 Performance Comparison

**FIGURE 2:**
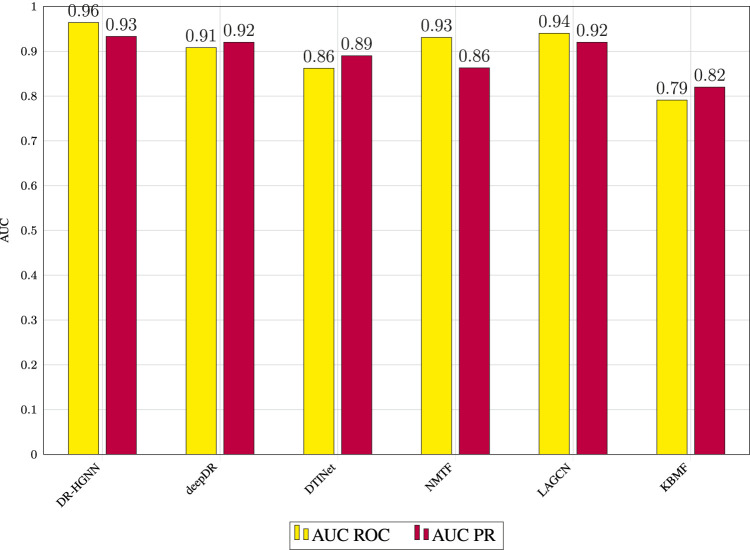
AUC ROC and AUC PR values of prediction results obtained by applying DR-HGNN and other reported methods in 5-fold cross-validation.

DR-HGNN’s results outperform all five methods on the DTINet data set. The parameters (that is, learning rate, dropout, optimizer function, number of layers, and embedding dimensions) in these methods are set to either their optimal values or recommended values reported in the original works. Figure 2 reports the AUC-ROC of all compared methods on the DTINet data. As shown in Figure 2, DR-HGNN outperforms other methods with 0.964 and 0.93 for its AUC-ROC and AUC-PR. An AUC-ROC and a loss history plot of DR-HGNN can also be seen in Figure 4 for both the training and validation datasets. Figure 2 also shows LAGCN with 0.94 for AUC-ROC, and 0.92 for AUC-PR is at the second place. deepDR also has the same AUC-PR value as LAGCN, but its AUC-ROC is around 0.91. NMTF as a matrix factorization-based method comes after neural network-based methods with 0.93 and 0.86 for its AUC-ROC and AUC-PR, respectively. Another matrix factorization-based method (KBMF) also has an AUC-ROC of 0.79 and AUC-PR of 0.82.

To illustrate the potential generalization of DR-HGNN, we evaluated another well-known benchmark dataset called the TL-HGBI dataset (Wang et al., 2014) with a 5-fold cross-validation (Table 3). The TL-HBGI dataset (Wang et al., 2014) has 1,409 drugs registered by the DrugBank database, 5,080 diseases listed by the OMIM database, and 1,461 known relationships. Drug–drug similarities were calculated based on their chemical structures, and a phenotype-based disease–disease similarity dataset was downloaded from MimMine. Table 3 shows that DR-HGNN outperforms other methods in both AUC and AUPR metrics. The results show that our approach can also compete with other methods with the AUC-ROC measure and the AUC-PR measure.

**TABLE 3:** Results of DR-HGNN on the TL-HBGI dataset (Wang et al., 2014).

Method	AUC	AUPR
TL-HGBI (Wang et al., 2014)	0.95	0.0492
NMF-DR (Sadeghi et al., 2021)	**0.9902**	0.4200
SCMFDD (Zhang et al., 2018)	0.97	0.1500
NTSIM (Zhang et al., 2017)	0.96	0.2631
DR-HGNN	0.9895	**0.4560**

### 3.4 Impact of Parameter Settings

**FIGURE 3:**
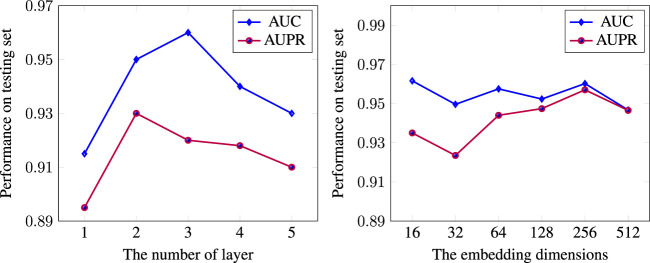
Impact of the number of layers and embedding dimension on the model performance.

We adjusted the parameters to achieve optimal performances. We showed the effect of using different learning rate parameters and dropout for the Adam optimizer in Table 4 with 50 epochs. Based on this experiment, the optimal learning rate and dropout for the Adam optimizer with *L*_2_ regularization was for 0.001 learning rate and 0.1 dropout. We also experimented with the size of the embedding. To investigate the effect of layer numbers on model performance, we compared results with a different number of layers in DR-HGNN on the DTINet dataset. Figure 3 showed the model performance along with the increase in layer numbers and embedding dimension.

**TABLE 4:** AUC ROC results for DR-HGNN based on different parameters.

Adam optimizer		Learning rate				
		**1.00E+00**	**1.00E-01**	**1.00E-02**	**1.00E-03**	**1.00E-04**
Dropout	0	0.8245	**0.9566**	0.9487	0.9639	0.8734
	0.1	0.8678	0.9165	0.954	**0.9647**	**0.8868**
	0.2	0.8605	0.9365	**0.9635**	0.8903	0.8778
	0.3	0.9333	0.916	0.9594	0.9167	0.8317
	0.4	0.898	0.9307	0.8955	0.937	0.827
	0.5	0.9492	0.9519	0.8986	0.9606	0.8319
	0.6	0.888	0.9469	0.892	0.9068	0.8687
	0.7	0.8682	0.8923	0.8682	0.961	0.7054
	0.8	**0.9501**	0.93	0.9106	0.9448	0.6968
	0.9	0.8922	0.9457	0.9357	0.9298	0.8472

**FIGURE 4:**
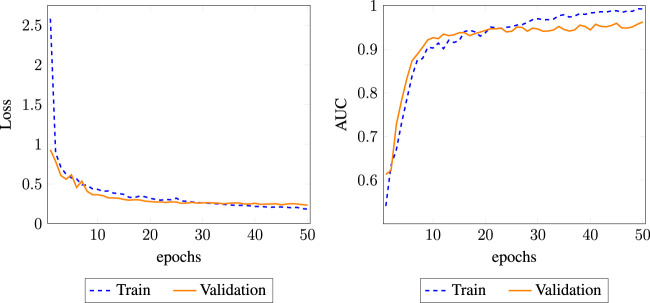
AUC and loss history plot for DR-HGNN on each epoch for the training and validation datasets.

We observed that one layer has the lowest performance, suggesting that a shallow network cannot sufficiently propagate the node feature to fuse heterogeneous information, especially for the complex DPD network. Moreover, we found that DR-HGNN achieved significant improvement with two layers’ structure. But with more than two layers, the model performance tends to decrease. We believe that GraphSAGE behaves similarly to graph convolutional networks (GCNs). A shallow GraphSAGE (1-layer) may not learn sufficient information, and more layers could lead to an over-smoothing issue. Other related works on the GNN also show that two layers are usually enough for capturing the knowledge of the network (Niu et al., 2021; Wang et al., 2020; Li et al., 2019; Chen et al., 2020). Figure 4 also shows the effect of embedding dimension on the DR-HGNN performance. Based on the results of this experiment, we chose the embedding dimension of 256 since the model has superior AUC-ROC and AUC-PR performance with this dimension size.

### 3.5 Case Study

In this section, we conducted case studies to evaluate the capability of DR-HGNN in predicting novel drug–disease associations. The relationships between drugs and diseases in the DTINet dataset not only have therapeutic ones but also have drug side effects. Thus, the model predicts both types of relations. Here, we discussed examples of both potential therapeutic relationships and potential side effects.

For verification of the prediction, along with a manual PubMed search, we have examined a publicly available Web server named ChemoText (Capuzzi et al., 2018). For example, hypertension (high blood pressure) has associations with the protein NADH dehydrogenase, subunit 1 (complex I) (UniprotId: P03886). Halothane (DrugBankID: DB01159), a general inhalation anesthetic used for the induction and maintenance of general anesthesia, has also been interacting with P03886. Chemotext and also Pubmed search also validate our connection between hypertension and halothane (Enderby, 1960). The other relationships we validated are between desflurane (DrugBank ID: DB01189), sevoflurane (DrugBank ID: DB01236), and pregabalin (DrugBank ID: DB00230) with hypertension and the shared protein of P03886. As for side effects, we validated nicotine (DrugBank ID: DB00184) associations. We found exciting relationships between nicotine with hyperalgesia. Nicotine is short-term pain relief; however, over time, it may increase pain intensity (Ditre et al., 2018). Nicotine also has an association with diabetic nephropathies and pregnancy complications through proteins P30926 and P36544, respectively.

## 4 Discussion and Conclusion

In this study, we presented a framework based on the GNN for drug repurposing. We created a heterogeneous drug–disease–protein network using multi-label problem transformation as input for heterogeneous GraphSAGE for repurposing drugs. Although we obtained satisfactory results, DR-HGNN has some limitations. First, we used several networks to create a heterogeneous drug repurposing network. However, in the future, we plan to consider more networks such as miRNA and genes to make a richer heterogeneous graph. Second, having multi-labeled edges in the drug–disease–protein network in the CDR task is a challenge that should be addressed. DR-HGNN uses the problem transformation approach for handling multi-label edges. MLC in drug repurposing has other challenges, such as label size imbalance. We can propose and use different solutions for this challenge in future work. All in all, DR-HGNN has the potential to be used for predicting edges in other biomedical networks, such as the drug–target interaction.

## Data Availability

The datasets analyzed for this study can be found in the DTINet GitHub repository (https://github.com/luoyunan/DTINet). The code for this study is available from the DR-HGNN repository (https://github.com/sshaghayeghs/DR_HGNN).
